# Heparin-Binding Haemagglutinin, a New Tool for the Detection of Latent *Mycobacterium tuberculosis* Infection in Hemodialysis Patients

**DOI:** 10.1371/journal.pone.0071088

**Published:** 2013-08-05

**Authors:** Rodrigue Dessein, Véronique Corbière, Joëlle Nortier, Max Dratwa, Karine Gastaldello, Agnieszka Pozdzik, Sophie Lecher, Bruno Grandbastien, Camille Locht, Françoise Mascart

**Affiliations:** 1 Laboratory of Vaccinology and Mucosal Immunity, Université Libre de Bruxelles, Brussels, Belgium; 2 Nephrology Department, Hôpital Erasme, Université Libre de Bruxelles, Brussels, Belgium; 3 Nephrology Department, Hôpital Brugmann, Université Libre de Bruxelles, Brussels, Belgium; 4 INSERM, Lille, France; 5 Institut Pasteur de Lille, Center for Infection and Immunity of Lille, Lille, France; 6 CNRS UMR8204, Lille, France; 7 Université Lille Nord de France, Lille, France; 8 Service de Gestion du Risque Infectieux, des Vigilances et d’Infectiologie, CHRU, Lille, France; 9 Immunobiology Clinic, Hôpital Erasme, Université Libre de Bruxelles, Brussels, Belgium; Johns Hopkins University School of Medicine, United States of America

## Abstract

**Background:**

Patients with end-stage renal disease (ESRD) and latently infected with *Mycobacterium tuberculosis* (LTBI) are at higher risk to develop tuberculosis (TB) than healthy subjects. Interferon-gamma release assays (IGRAs) were reported to be more sensitive than tuberculin skin tests for the detection of infected individuals in dialysis patients.

**Methods:**

On 143 dialysis patients prospectively enrolled, we compared the results from the QuantiFERON®-TB Gold assay (QFT), to those of an IGRA in response to *in vitro* stimulation of circulating mononuclear cells with the mycobacterial latency antigen Heparin-Binding Haemagglutinin purified from *Mycobacterium bovis* BCG (native HBHA, nHBHA).

**Results:**

Seven patients had a past history of active TB and 1 had an undetermined result with both IGRAs. Among the other 135 patients, 94 had concordant results with the QFT and nHBHA-IGRA, 40.0% being negative and therefore not latently infected, and 29.6% being positive and thus LTBI. Discrepant results between these tests were found for 36 patients positive only with the nHBHA-IGRA and 5 only with the QFT.

**Conclusions:**

The nHBHA-IGRA is more sensitive than the QFT for the detection of LTBI dialysis patients, and follow-up of the patients will allow us to define the clinical significance of discrepant results between the nHBHA-IGRA and the QFT.

## Introduction

Tuberculosis (TB) remains a major public health problem both in developing and industrialized countries. One third of the world population is considered infected with *Mycobacterium tuberculosis* (*Mtb*), and 8.8 million new cases of active TB were reported in 2010 causing 1.4 million deaths [1]. Fortunately most infected subjects do not develop symptoms of disease during their lifetime. They maintain a latent *Mtb* infection (LTBI) but remain lifelong at risk to develop the disease [2], the highest risk of progression to active disease being within the first two years after infection, or at any time in a context of immunosuppression. They represent therefore an important reservoir of possible future active TB, so that the identification and preventive treatment of these individuals are key elements in TB control [3], [4]. The detection of LTBI subjects currently relies on the detection of an immune response to mycobacterial antigens. Whereas tuberculin skin tests (TST) have long been the only method to detect such immune responses, *in vitro* tests based on the release of interferon-gamma (IFN-γ) (interferon-gamma release assay, IGRA) by the blood cells in the presence of mycobacterial antigens have more recently been developed. Two such tests are commercially available, the QuantiFERON®-TB Gold In-Tube (QFT) (Cellestis Ltd, Carnegie, Australia) and the T-SPOT®.TB (Oxford Immunotec, UK). Compared to TST, these commercialized tests clearly improve the detection of LTBI among patients under immunosuppressive therapy [5], [6], and they provide better specificity than the TST among BCG-vaccinated populations.

Patients with end-stage renal disease (ESRD) treated by iterative hemodialysis (HD) are a group of patients for which detection of LTBI is highly recommended [7], as, depending of the country where they are living, they have a 10–25 fold higher risk than healthy subjects to reactivate the *Mtb* infection and to develop active TB [8]–[14], as a result of underlying immune dysfunction [15]. In addition, they have a higher risk to be infected as they are regularly exposed to patients with active TB in HD centres [16], and a recent prospective study reported a surprisingly high prevalence of LTBI among ESRD patients [17]. The risk of active TB in these patients is even increased 50-fold for those who will be treated with transplantation [13], and the nonspecific presentation of these cases of active TB often leading to delayed diagnosis is well-known by nephrologists [7], [14]. As the sensitivity of TST for the detection of LTBI among HD is poor [7], [18]–[22], commercialized IGRAs have been evaluated by several groups, but only a limited number of these studies contain enough data to be included in a recent meta-analysis indicating that, compared to TST, these IGRAs are associated more strongly with risk factors for LTBI [23]. Most studies were performed on 30 to 60 patients and reported an incidence of 25% to 47% of LTBI among HD patients in low and intermediate TB incidence countries, respectively [16], [17], [20], [24]–[27].

However, recent data indicated that the commercially available IGRAs are not optimal for the detection of all LTBI subjects [28]–[30], remaining imperfect for the detection of LTBI among ESRD as they leave false-negative results [16]. In addition, a high number of undetermined results was reported with these tests as well as a poor detection of patients with a previous history of TB [16], [19]. In fact, these commercialized IGRAs preferentially identify recently infected subjects [18], [31], [32], and a better detection method of LTBI, including those resulting from an old infection, may be offered by IGRAs based on latency antigens, such as the native Heparin-Binding Hemagglutinin (nHBHA) [28], [30] or DosR-regulon-encoded antigens [32]. Such a screening for all LTBI subjects probably provides only little benefit among immune-competent individuals with specific immune responses maintaining long-term immune control of the infection. In contrast, a decrease of these responses may occur at any time in immune-compromised subjects like HD patients leading to the progression to active TB so that these patients should be detected to benefit from a prophylactic treatment [13].

In this study, we performed a prospective cross-sectional study to compare the QFT to a nHBHA-IGRA for the detection of LTBI in 143 patients with ESRD treated by iterative HD. The *in vivo* responses to Tuberculin Purified Protein Derivative (PPD) were evaluated as the TST still represent a gold standard for the detection of LTBI and the *in vitro* responses to PPD, even if lacking specificity for *Mtb* infection, were tested in parallel as they are more sensitive than TST in HD patients [22].

## Materials and Methods

### Patients

Hundred forty three patients suffering from ESRD with a residual creatinine clearance <10 ml/min and treated by HD were prospectively enrolled. The exclusion criteria were the hemodynamic instability, the transplanted or pharmacologically immuno-compromised patients. Beside ESRD other TB risk factors, such as malnutrition, diabetes, HIV infection, AIDS, tobacco addiction, chronic obstructive pulmonary disease were retrospectively extracted from medical records as well as the age, country of origin, and the results of the PPD-TST performed as part of the evaluation of HD patients. The protocol (P2008/344) was approved by “the Ethics Committee of Erasme Hospital” (N°OMO21) and all the patients gave written informed consent before enrolment.

### Immune Response to Purified Protein Derivative (PPD)

TST is the recommended test for detection of LTBI in Belgium and was therefore performed as part of the evaluation of the patients, except for those with a past-history of active TB as TST should never been repeated if it has previously been positive [14]. The standard TST procedure in Belgium is performed by injecting 2 PPD RT23 units (Staten Serum Institute, Denmark) intradermally on the palmar aspect of the forearm, and by measuring the diameter of the skin induration 72 hrs later. A positive result is defined by the diameter of the skin induration related to the clinical risk of the patient as defined by the CDC [33], or by a conversion of the skin test defined as a change of the induration diameter of more than 10 mm between two TST. For HD patients, TST is considered positive for values higher than 10 mm in view of the known immune-depression in these patients [22], and a two-step TST is not recommended. Diagnosis of latent TB is retained in the presence of a positive TST together with a negative chest X-ray, negative microbiological tests, and absence of clinical symptoms.

In view of the reported poor sensitivity of TST in HD patients [7], [18]–[22], the *in vitro* immune response to PPD was also evaluated by an IGRA performed as previously reported on 96 hrs *in vitro* PPD-stimulated peripheral blood mononuclear cells (PBMC) [28]. The cut-off determined by Receiver Operating Characteristic (ROC) curves established on results from non-infected controls and healthy LTBI subjects was fixed at 500 pg/ml of IFN-γ to reach 98.5% sensitivity and to represent an index of exposure to mycobacteria.

### QuantiFERON®-TB Gold In-Tube

QFT assays were not part of the routine evaluation to detect LTBI in Belgium. They were performed according to the manufacturer’s recommendations (www.cellestis.com). Plasma IFN-γ concentrations were measured after 24 hrs incubation at 37°C, and the results were recorded as positive, negative or undetermined (IFN-γ concentrations lower than 0.5 UI/ml in response to polyclonal stimulation).

### nHBHA-IGRAs

An HBHA-IGRA performed on PBMC *in vitro* stimulated with native methylated HBHA (nHBHA-IGRA) during 96 hrs was previously reported as being able to detect LTBI among healthy subjects with 92% sensitivity and 94% specificity [28]. We developed here a short incubation time (24hrs) nHBHA-IGRA to allow better comparison with the QFT suggested to detect IFN-γ secreted by mostly effector memory T cells responses whereas longer incubation time IGRA probably detect IFN-γ secreted by both effector memory and central memory T cell responses [29]. PBMC were purified from fresh venous blood samples collected before starting the dialysis session [34], and they were adjusted to 2.10^6^/ml in culture medium. Native methylated HBHA was purified from *M. bovis* BCG as detailed elsewhere [35], [36], and added to the PBMC at 2 µg/ml [28]. As suggested by results obtained for other assays of cellular immune responses, interleukin-7 (IL-7) (5 ng/ml, R&D Systems) was added to the culture medium to raise the sensitivity of the assay [37]. Cell culture supernatants were collected after 24 hrs incubation at 37°C, and the IFN-γ concentrations were measured by the IFN-γ cytoset ELISA (BioSource International, Camarillo, CA, USA) as previously described [28]. The effect of IL-7 (5 ng/ml) on the assay sensitivity without any loss in specificity was evaluated by measuring the IFN-γ concentrations released by the PBMC from 7 healthy LTBI subjects and from 7 healthy controls in the absence or presence of nHBHA (2 µg/ml). In addition, the results obtained with the 24 hrs nHBHA-IGRA assay were compared to those obtained with the previously described 96 hrs nHBHA-IGRA for 17 healthy LTBI and 12 healthy non-infected subjects (5/12 were BCG vaccinated) as well as for 96 HD patients. Healthy LTBI subjects were selected among health care workers on the basis of a known and objectivised conversion of their TST and healthy controls were selected on the basis of a negative TST. Non-stimulated PBMC and PHA-stimulated PBMC were also incubated for 24 hrs at 37°C as negative and positive controls, respectively. Except for the assays on the effect of IL-7 on the IFN-γ secretion, results obtained for non-stimulated cells were subtracted from those obtained in response to the stimulation with antigens or mitogen.

### Statistical Analyses

Correlation between the 96hrs and the 24hrs nHBHA-IGRA was analyzed by non-parametric Spearman test and differences between assays were assessed by the non-parametric Mann-Whitney test. Statistical analysis was performed with the Graphpad Prism Software version 4.0b (San Diego, CA, USA, www.graphpad.com). Results were expressed as percentages for categorical variables and as medians and interquartile ranges for numerical variables. The Mann-Whitney test was used for comparisons of continuous variables and Chi-square or Fisher’s exact tests for categorical variables. Multivariate logistic regressions were performed to analyze independent risk factors, applying Hosmer-Lemeshow goodness of fit test [38]. A value of p<0.05 was considered to be significant. Adjusted odds ratios (aOR) with their 95% confidence intervals (CI) were calculated with estimated regression coefficients and their standard errors in the logistic regression analysis. The agreement between IGRAs was evaluated by kappa statistic considering values between 0.40 and 0.75 as fair to good agreement [39]. SPSS software, version 15.0, was used for these statistical analyses.

## Results

### Clinical and Demographic Characteristics of the Patients

Medical records revealed that 7 patients had developed TB in the past so that they were excluded from further analysis concerning LTBI detection. No sign of active or past TB was noted on the chest X-ray from the remaining 136 patients who had also no clinical or biological sign of current infectious diseases at the time of blood testing. Their BCG status was unknown as most patients were unable to remember if they were vaccinated in the past. Sixty-nine patients originated from a high TB prevalence country (33 from Western Africa, 24 from North Africa, and 12 from Eastern Europe). Compared to patients from a low TB prevalence country, they were younger and had less medical TB risk factors but hypertensive and diabetic nephropathies were more frequently recorded as the ESRD cause (Table 1). Patients originating from Eastern Europe most often arrived in Belgium less than one year before inclusion whereas those originating from Africa were most often living in Belgium since several years. However, patients from North Africa usually go back to their country of origin once every year, being therefore possibly re-exposed to *Mtb*. None of the included patient reported a previous prophylactic treatment against TB.

**Table 1:** Demographic and clinical data of the HD patients a.

	Low TB Prevalence	High TB Prevalence	Univariate analysis b	Multivariate analysis	
	n	n	p value	p value	aOR (95%CI) c
Number of inclusion	67	69			
Age (years) d	66 (54–71)	59 (45–71)	0.046	0.007	0.96 (0.94–0.99)
Sex Ratio F/M	25/42	23/46	0.627		
Number of tuberculosis risk factors d,e	2 (1–4)	1 (1–5)	0.0006	0.001	0.45 (0.28–0.72)
Time on dialysis (yrs) d	5 (0.62–9.6)	3 (0.61–9.2)	0.029		
End stage renal disease etiology					
-Hypertensive nephropathy	15	28	0.027	0.036	2.57 (1.06–6.23)
-Diabetic nephropathy	14	26	0.037	0.001	5.65 (2.10–15.2)
-Glomerulonephritis	10	9	0.726	0.567	1.39 (0.45–4.27)
-Interstitial nephritis	9	11	0.707		
-Vascular surgery	2	1	0.535		
-Polycystic kidneys	7	1	0.025		
-Vascular renal disease	5	1	0.085		
-Infection	3	3	0.956		
-Malignancy	3	0	0.074		
-Unknown	4	9	1		

a After exclusion of the patients with a TB history; one patient from the low TB prevalence group was further.

excluded for undetermined results obtained in the IGRAs.

b Mann-Whitney U or Chi-square test.

c aOR (95%CI) : Adjusted Odds ratio and 95% confidence interval.

d Median (interquartile range).

e Tuberculosis risk factors: end stage renal disease, malnutrition, diabetes, HIV infection, AIDS, tobacco addiction, chronic obstructive pulmonary disease.

Final multivariate model: Hosmer-Lemeshow goodness of fit test, p = 0.11.

### *In vivo* and *in vitro* Immune Responses to Purified Protein Derivative (PPD)

Results from TST were recorded for 115 patients from whom only 4 developed an induration >10 mm, and 10 displayed an induration between 2–6 mm.

In contrast, the PPD-IGRA was positive for 66/136 (48.5%) of the patients globally and for 66/115 (57.4%) of those who underwent a TST, indicating the absence of agreement between the TST results and those of the PPD-IGRA in HD patients. One PPD-IGRA result was undetermined, whereas the test was positive for all the patients with a past TB-history (median IFN-γ concentration: 1,985 pg/ml; range: 545–9,140). A strong association was noted between the PPD-IGRA positivity and male gender (OR 4.42, 95% CI: 1.85–10.52).

### QFT Results

**Figure 1:**
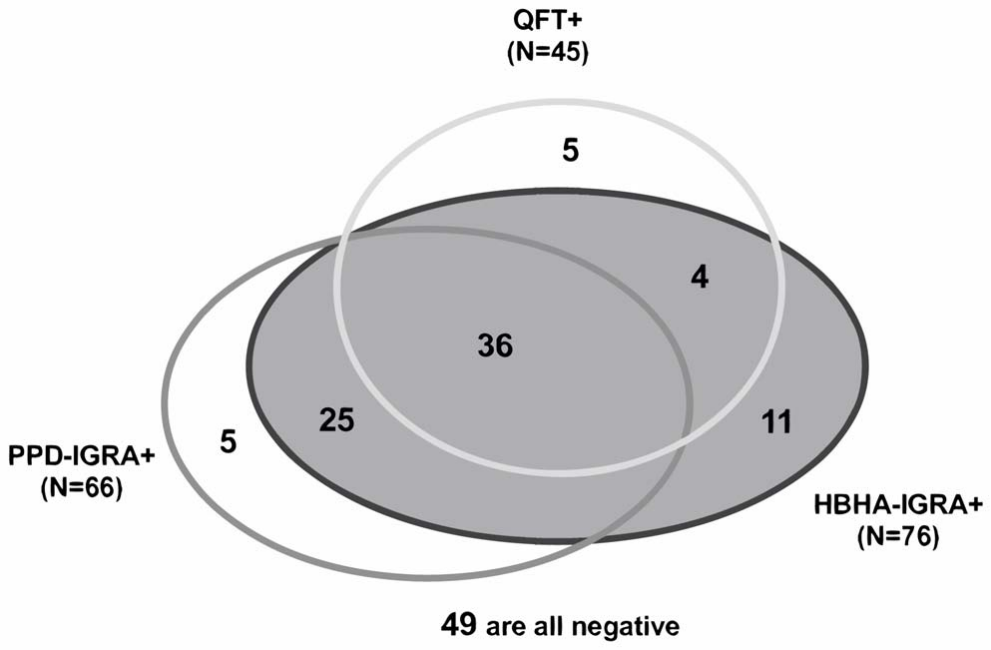
Venn diagram illustrating the positive IGRAs in ESRD patients. The different IGRAs are depicted by three circles within which are included the number of positive assays. In the case of positive patients for two or three different assays, they are represented in the intersections of the circles.

The QFT was positive for 4/7 patients with a past history of TB. After exclusion of the only patient with an undetermined result from the remaining patients, a positive QFT was obtained for 45/135 patients (33.3%) (Figure 1). Only one of them had a positive TST. Multivariate logistic regression analysis indicated an association between greater age (OR 1.04, Table 2), and male gender (OR 2.92, Table 2) with a positive QFT whereas the number of TB risk factors and ESRD origins did not influence the positivity of the QFT. The QFT was positive for 28/69 (41%) and 17/66 (26%) of the patients from high and low TB-incidence countries, respectively. This means that 62.2% of the patients with a positive QFT in this study were born in a high TB prevalence country compared to 37.8% born in Western countries. Compared to Western Europe, the highest risk for a positive QFT among high-TB prevalence countries was for patients originating from North Africa (OR 4.96, Table 2).

**Table 2:** Multivariate logistic regression model and odds ratio for positive tests in HD patients.

	QuantiFERON-TB Gold		nHBHA-IGRA	
	aOR*95%CI)	p value	aOR*(95%CI)	p value
Age	1.04 (1.01–1.07)	0.014	NS	
Gender (M vs F)	2.92 (1.19–7.14)	0.020	2.60 (1.14–5.88)	0.023
Nb of TB risk factors	NS		NS	
ESRD etiology	NS		NS	
Country of origin				
-Western Europe (N = 66)	Reference		Reference	
-Western Africa (N = 33)	1.38 (0.46–4.16)	0.567	3.51 (1.28–9.63)	0.015
-Eastern Europe (N = 12)	0.72 (0.14–3.82)	0.700	9.93 (1.94–50.90)	0.006
-North Africa (N = 24)	4.96 (1.91–12.91)	0.001	9.48 (3.30–27.30)	<10^−3^

* aOR (95%CI): Adjusted Odds ratio and 95% confidence interval; NS: Not Significant.

### Validation of the 24 hrs nHBHA-IGRA

**Figure 2:**
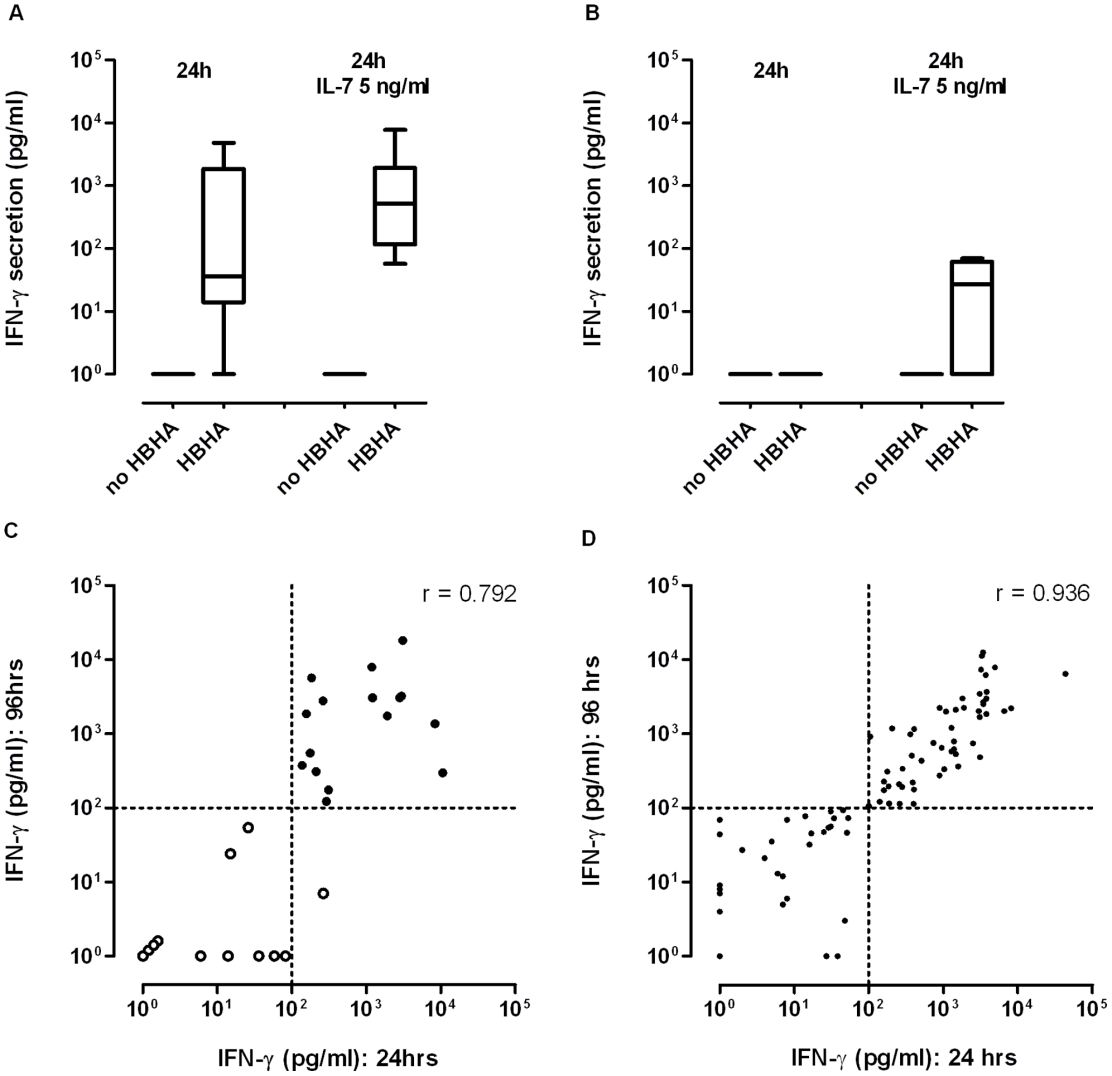
Validation of a 24hrs nHBHA-IGRA. The effect of IL-7 on the IFN-γ secretion was analysed for 7 LTBI (panel A) and 7 control subjects (panel B). PBMC were *in vitro* incubated during 24 hrs with or without 2 µg/ml of nHBHA in the absence or presence of 5 ng/ml IL-7. The IFN-γ concentrations released in the cell culture supernatants were measured by ELISA. Box and whiskers represents the median, 25^th^–75th percentiles, and the ranges of IFN-γ concentrations obtained for the included subjects. The results from the 24 hrs and the 96 hrs nHBHA-IGRA were compared for 29 immunocompetent subjects (panel C) and for 96 haemodialysis patients (panel D). PBMC were *in vitro* incubated during 24 hrs in presence of 2 µg/ml nHBHA with 5 ng/ml IL-7 or during 96 hrs in presence of 2 µg/ml nHBHA without IL-7, for 12 controls and 17 LTBI individuals (panel C, open and black circles respectively), and for haemodialysis patients (panel D). Correlations between IFN-γ concentrations released in the cell culture supernatants obtained with the two different incubation times are represented on the figure, each dot corresponding to a single subject. The values of the r Spearman correlation index are 0.792 (p<0.0001) and 0.936 (p<0.0001) on panel C and D, respectively.

The addition of 5 ng/ml IL-7 to the culture medium increased the concentrations of IFN-γ released by the PBMC after 24 hrs of culture in the presence of nHBHA, with IFN-γ concentrations being significantly higher for LTBI compared to control subjects (p = 0.002) (Figure 2, panel A–B). No rise in the IFN-γ concentrations released by the PBMC in culture medium supplemented with IL-7 without antigen was observed (Figure 2, panel A–B).

To further validate the 24 hrs nHBHA-IGRA, IFN-γ released concentrations were measured for 12 healthy non-infected controls and 17 healthy LTBI individuals, both after 24 hrs of culture in the presence of IL-7 and after 96 hrs incubation without IL-7. Results obtained with both incubation times were well correlated as shown in Figure 2C (r Spearman index = 0.792, p<0001). Based on this comparison, it was clear that the cut-off value of 100 pg/ml IFN-γ previously determined for the 96 hrs nHBHA-IGRA [28] was also appropriate for the 24 hrs IGRA, all LTBI subjects being identified with both tests. A comparison between the two assays was also performed for the 96 first HD patients included. As shown in Figure 2D, both incubation times were perfectly correlated (r Spearman index = 0.936, p<0001). Undetermined results were defined as an IFN-γ concentration higher than 100 pg/ml or lower than 200 pg/ml for non-stimulated and polyclonaly-stimulated PBMC respectively.

### 24hrs nHBHA-IGRA Results

**Figure 3:**
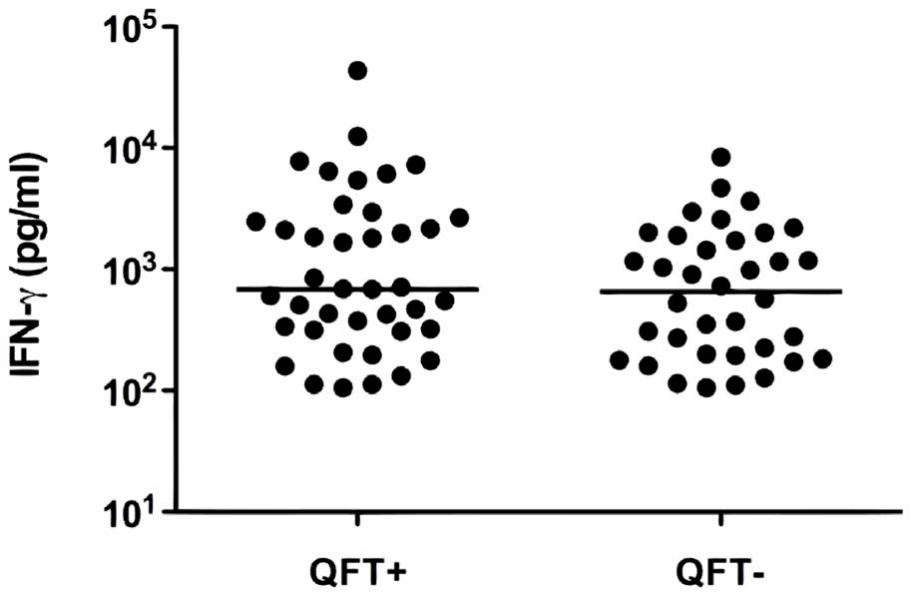
Positive nHBHA-IGRA results in QFT positive or negative HD patients. PBMC freshly isolated from whole blood of 135 HD patients were incubated 24 hrs in presence of nHBHA (2 µg/ml) with 5 ng/ml IL-7, and the IFN-γ concentrations released in the cell cultures supernatants were measured by ELISA. Seventy-six subjects were nHBHA-IGRA positive. QFT was performed simultaneously and each dot represents a positive nHBHA-IGRA result (expressed in pg/ml) for single patient presenting either a positive QFT (QFT+ = 40) or a negative QFT (QFT− = 36). Horizontal bars represent the medians of the results.

The nHBHA-IGRA was positive for the 7 patients with a previous history of active TB. Among the others, one patient had an undetermined result for the nHBHA-IGRA like for the QFT, and 76 had a positive test (56.3%), with a median value of IFN-γ release of 687 pg/ml (range: 106–43,322). Among these, 40 had also a positive QFT (Figure 1), and the nHBHA-induced IFN-γ concentrations were similar in the QFT positive compared to the QFT negative group (Figure 3). As for the QFT results, a weak association was found between the nHBHA-IGRA positivity and the male gender (OR 2.60, Table 2). The nHBHA-IGRA was positive for 53/69 (77%) and 23/66 (35%) of the patients from high and low TB-incidence countries, respectively. This means that among the patients with a positive nHBHA-IGRA, 69.7% were born in a high prevalence country, compared to 30.3% born in Western countries. Strong associations were noted between the positivity of the nHBHA-IGRA and the country of origin (OR 9.93 for Eastern Europe, OR 9.48 for North Africa, and OR 3.51 for Western Africa, Table 2). In contrast, no association was observed between ESRD etiologies and the nHBHA-IGRA positivity.

### Comparison of the PPD-IGRA, QFT and nHBHA-IGRA in HD Patients

Globally, concordance between the tests assessed using *k* coefficients for the 135 patients who had no past history of TB and no undetermined results indicated a good concordance between the different IGRAs with a *k* coefficient of 0.705 (95% CI: 0.587–0.823) between the nHBHA- and the PPD-IGRA, 0.417 (95% CI: 0.282–0.552) between the nHBHA-IGRA and the QFT, and 0.418 (95% CI: 0.271–0.565) between the PPD-IGRA and the QFT.

**Figure 4:**
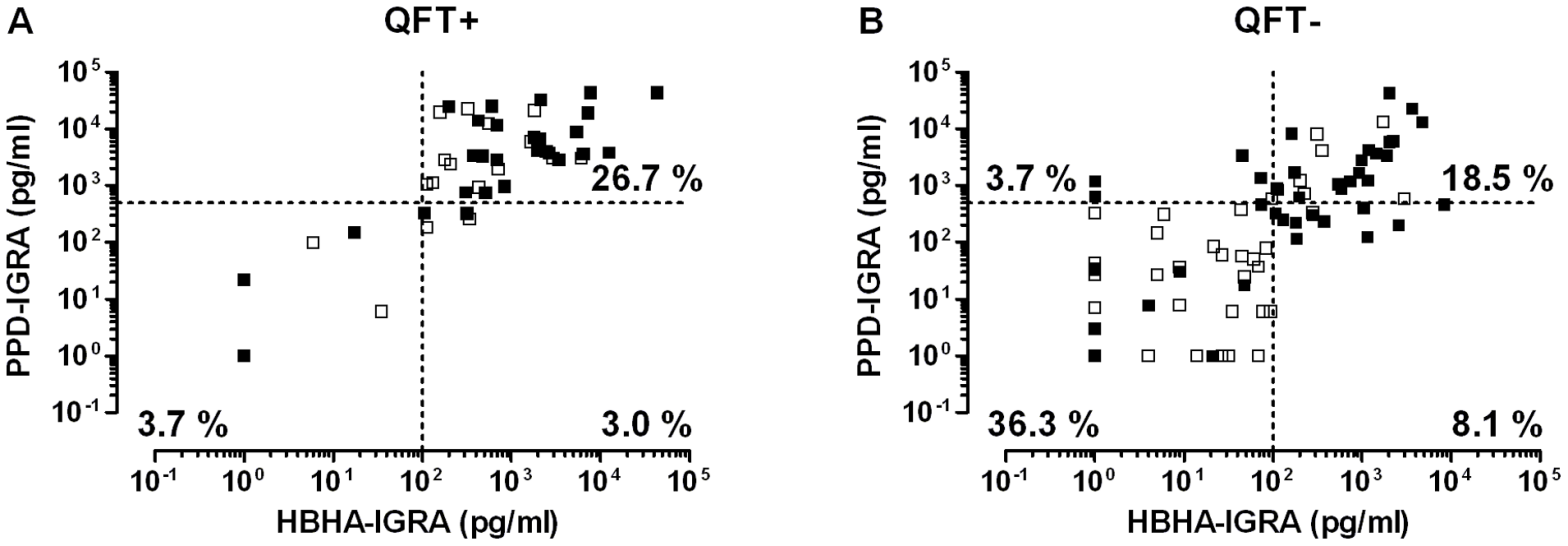
Comparison of the different IGRA results obtained in HD patients. PBMC freshly isolated from whole blood of 135 HD patients were incubated 24hrs in presence of nHBHA (2 µg/ml) with 5 ng/ml IL-7, or 96hrs in presence of PPD (4 µg/ml) before measuring the corresponding IFN-γ concentrations released in the supernatant. The squares represent the IFN-γ concentrations (pg/ml) in response to PPD and nHBHA in QFT positive patients (Panel A, n = 45) and in QFT negative patients (Panel B, n = 90). Each square symbolizes a single patient, black and open squares representing patients originated from high and low incidence TB countries, respectively. Dotted lines indicate the positivity cut-off for each test. Percentages represent the proportion of results within the indicated quadrant among the total number of patients (n = 135).

For a detailed comparison of the results, the 135 patients were divided in two groups based on the results of the QFT test, positive or negative (Figure 4). Among the patients with a positive QFT test (n = 45), 62.2% originated from a high TB-incidence country as illustrated on Figure 4A. In this group with positive QFT, 36 patients out of 45 were also positive for the nHBHA- and for the PPD-IGRA, suggesting that 26.7% of the 135 patients were undoubtfully *Mtb* infected (Figure 1 and 4A). Five patients had an isolated QFT test associated with very low responses to PPD (less than 200 pg/ml) and to nHBHA (less than 50 pg/ml) (3.7% of the 135 patients). Three out of five could be retested 12 months later with one of them being totally negative and the other two patients having still a positive QFT with no response to PPD or nHBHA. The significance of these results remains unknown. Four patients were positive with the QFT and the nHBHA-IGRA but were slightly below the cut-off value for the PPD-IGRA (Figure 4A), indicating that this cut-off established on healthy infected subjects, may perhaps be slightly too high for the HD patients with diminished cellular immune responses [21].

Among the patients with a negative QFT test (n = 90), 45.6% originated from a high TB-incidence country as illustrated on Figure 4B. In this group with negative QFT, 49 patients out of 90 were also negative for both the nHBHA- and the PPD-IGRA identifying the group of patients who were not *Mtb* infected (36.3% of the 135 patients) (Figure 4B). Five patients were only positive for the PPD-IGRA, which may perhaps be related to the poor specificity of the PPD-IGRA. In contrast, among the PPD positive patients, 25 were also positive for the nHBHA-IGRA (18.5% of the included patients) suggesting, based on previously reported data [28], [30], that they presented with latent TB, even though the QFT test was negative (Figure 1 and 4B). Eleven patients were only positive for the nHBHA-IGRA but for 9 of them, the PPD-IGRA values were again only slightly below the cut-off value (between 200 and 500 pg/ml IFN-γ) confirming this cut-off was inappropriate for HD patients. Ten out of these 11 patients originated from a high TB incidence country, being therefore more at risk to be *Mtb*-infected. Comparing by ROC curve analysis the IFN-γ concentrations of the PPD-IGRA from patients with an undoubtfully latent TB (QFT and nHBHA-IGRA positive) with those from clearly non-infected patients (QFT and nHBHA-IGRA negative), the optimal cut-off value of 200 pg/ml IFN-γ was found to provide a sensitivity of 98% of the test.

Based on this adapted cut-off of the PPD-IGRA, 25.2% of the 135 patients had positive nHBHA- and PPD-IGRAs but a negative QFT test whereas 29.6% of them had a positive QFT associated with nHBHA- and PPD-positive IGRAs.

## Discussion

Detection of LTBI in HD is recommended by different authors, even if current CDC guidelines still do not recommend screening patients for LTBI except if they are kidney transplant candidates [7], [14], [18]. Using a Markov model for screening and preventive treatment for LTBI among HD patients, Rose estimated that only between 140 and 5,000 patients would have to be screened to prevent one case of TB [13]. However, the poor sensitivity of the TST to detect LTBI among HD patients is well known [7], [18]–[22], as well as the imperfection of the new IGRAs that provide false-negative results [16], [28]–[30].

In this paper, we report the results obtained for 143 HD patients living in a low TB prevalence country by combining two different IGRAs, the commercial QFT and a home-made IGRA using a latency antigen, the nHBHA. Seven patients had a past history of active TB, and they all had a positive nHBHA-IGRA, whereas the QFT was positive for only 4 of them confirming the relative sensitivity of a commercial IGRA to detect previous TB infection [16]. Moreover, we confirmed the higher sensitivity of the QFT compared to TST to detect LTBI among HD patients as the QFT was positive for 33.3% of the patients compared to only 3.5% of positive TST results. Undetermined results were not a limitation in this study (only 1/143), in contrast to many reported studies but confirming Segall’s report [7]. We reported here a very low sensitivity of the TST compared to other studies [16], [17], [20] and these differences may be due to the TST procedure recommended in Belgium (one step procedure with 2 PPD units).

However, the data obtained with the nHBHA-IGRA confirmed that the proportion of LTBI HD patients detected with the QFT is underestimated, as 56.3% of the HD patients of our series were detected as LTBI with the nHBHA-IGRA. We previously reported the value of the nHBHA-IGRA to detect LTBI among healthy subjects with evidences suggesting its higher sensitivity compared to the QFT, especially for LTBI subjects with a stable and controlled LTBI [28], [30]. We report here for the first time the sensitivity of the nHBHA-IGRA to detect LTBI among immuno-suppressed patients, this IGRA allowing us to maximize the sensitivity of LTBI detection in HD patients in order to correctly identify as many truly infected patients as possible. One could argue that at least some of the positive results for the nHBHA-IGRA could be secondary to a previous BCG vaccination of some patients or to a possible cross-reactivity with HBHA homologues from non typable mycobacteria (NTM) that could have infected HD patients originating from high TB prevalence countries. The possible influence of a previous BCG vaccination on the results of the nHBHA-IGRA was previously carefully analyzed and we reported that an old BCG vaccination (more than 15 years) had no influence [28]. We cannot totally exclude a possible cross-reaction with NTM but if it was the case, it would only represent a marginal proportion of the positive patients. As we showed that the immunogenicity of nHBHA is strictly dependent on the methylation pattern of nHBHA [40], the number of possible cross-reaction should be quite low.

The higher sensitivity of the nHBHA-IGRA compared to the QFT to detect LTBI among HD patients is consistent with the current literature. The QFT was reported to be especially suitable for the detection of recent *Mtb* exposure in HD patients [18], a clinically important issue because of the well-known risk of *Mtb* transmission in dialysis facilities [7], [14]. In contrast, its sensitivity to detect among HD patients LTBI resulting from an old infection and at risk to reactivate the infection is not clearly established. Some degree of consensus appears in the current literature about the advantage of using antigens encoded by the RD-1 portion of the *Mtb* genome to detect recent *Mtb* infection, and to use latency antigens for the detection of LTBI resulting from past infections [31], [32]. However, in the context of the recently reviewed concept of LTBI [2], the significance for a patient to be positive in both tests (QFT and nHBHA-IGRA) compared to only nHBHA-IGRA positive may be different [30]. Since nHBHA is a protective antigen [41], an IFN-γ response to nHBHA may be considered as a correlate for protection, consistent with the fact that it is more often detected in subjects with LTBI than in those with active TB [28]. In contrast, the IFN-γ responses to ESAT-6, one of the QFT antigens, have been suggested to be risk factor for the development of active TB [31], [42]. Among the 28 patients included here who were followed at least for one year, two of them who initially had a positive nHBHA-IGRA developed active TB when their IFN-γ response to nHBHA became barely detectable and was associated with a positive QFT [30]. Two other patients not included in this study initially and with a high clinical suspicion of active TB had a positive QFT with a negative nHBHA-IGRA. Long-term follow-up studies of HD patients will help to solve this question.

The prevalence of LTBI detected here in HD patients originating from and living in Western Europe was 26% with the QFT, which is similar to that reported in Switzerland [24], and 35% with the nHBHA-IGRA. As discussed above, the difference between the two tests most likely allows identifying patients with an old infection that probably represent numerous patients in our Belgian cohort with a high median age (Table 1), and therefore possible *Mtb* infection during childhood when even in Belgium the TB prevalence was high. However, as detection of LTBI in HD patients is mostly recommended if treatment is planned [14], and as preventive therapy may not be useful in patients with short life expectancies in view of the high risk of adverse effects, screening for LTBI in our HD patients living in Belgium should be limited to those who are young enough to be waiting for an eventual graft.

The nHBHA-IGRA was more often positive for the three groups of patients originating from high TB prevalence countries, compared to patients from low TB prevalence countries, whereas the QFT was more often positive only for patients originating from North Africa and essentially travelling back there at least once a year and being possibly re-infected. Accurate detection of LTBI among HD patients originating from high TB prevalence countries and living now in low prevalence countries, where they are treated, may be important in view of their high risk to be LTBI and of their risk of *Mtb* reactivation. These patients are most often still young (median age, 59 years) with therefore reasonable life expectancy. Some are waiting for a possible graft, and for all of them, rapid diagnosis of a *Mtb* reactivation is important for an early appropriate treatment. In these patients, even if a prophylactic treatment of LTBI is not always provided, knowing they are LTBI will help to get a prompt diagnosis in case of clinical deterioration especially in case of modification of their IGRAs profile [30].

We conclude that IGRAs are quite helpful for the detection of LTBI among ESRD patients, essentially among those who will be grafted and for whom a prophylactic treatment of LTBI should therefore be undertaken [33]. Additionally, we suggest using IGRAs for LTBI detection in those patients with a high underlying risk to have LTBI and with a reasonable life expectancy. In these patients, IGRAs should be used as part of a comprehensive risk assessment in view of their high risk for TB morbidity and mortality. Decision of prophylactic chemotherapy should be taken on an individual basis, depending of the degree of immuno-suppression of the patient and the number of morbidity factors. In addition, combining the results from two different IGRAs, the QFT and the nHBHA-IGRA, allows us to detect LTBI among HD patients with a high sensitivity, may help to stratify the patient’s risk of *Mtb* reactivation, and may even suggest the development of the reactivation.
